# Quantitative Optical Coherence Elastography for Robust Stiffness Assessment

**DOI:** 10.3390/app8081255

**Published:** 2018-07-30

**Authors:** Xuan Liu, Farzana Zaki, Yahui Wang

**Affiliations:** Department of Electrical and Computer Engineering, New Jersey Institute of Technology, University Heights, Newark, NJ 07102, USA

**Keywords:** optical coherence tomography, optical coherence elastography, optical sensing and sensors, tissue characterization

## Abstract

We demonstrated the capability of quantitative optical coherence elastography (qOCE) for robust assessment of material stiffness under different boundary conditions using the reaction force and displacement field established in the sample.

## 1. Introduction

Breast conserving surgery is one of the most frequently practiced surgical procedures for the treatment of breast cancer. In breast conserving surgery, a negative surgical margin is essential to reduce the risk of local recurrence and reduce the need for repeated surgery. The capability to assess surgical margin intraoperatively can benefit both patients and clinicians [1]. It is known that cancerous breast tissue usually has higher stiffness compared to normal breast tissue [2]. Therefore, manual palpation is frequently used in clinical examination of breast cancer. Elastography techniques based on cross-sectional imaging modalities, such as ultrasound imaging and magnetic resonance imaging (MRI), have also been used for breast imaging [3,4]. Optical coherence tomography (OCT), a microscopic tomographic imaging modality based on low coherence light interferometry, has found applications in breast cancer management [5–8]. A functional extension of OCT, optical coherence elastography (OCE) provides mechanical contrast and can be used to differentiate cancerous breast tissue and normal breast tissue [9–11]. Compared to other elastography technologies, OCE has much higher spatial resolution and allows mechanical characterization on a small volume of breast tissue. An OCE instrument can be fabricated using fiber-optic components and can be integrated into a handheld probe that is compact and lightweight [12]. Therefore, an OCE instrument will allow convenient assessment of tissue malignancy for margin assessment during breast conserving surgery, and has the potential to significantly improve patient outcome by ensuring negative margin. However, conventional OCE that only tracks the deformation of the sample is inherently qualitative because the reaction force considered as the mechanical excitation is unknown. Therefore, the results obtained from different OCE measurement sessions can vary significantly for the same sample, making it challenging to establish reliable criteria for tissue classification. In our laboratory, we recently developed and validated a unique fiber-optic quantitative OCE (qOCE) technology [13,14]. For mechanical characterization, the qOCE probe is translated to compress the sample. The OCT signal is obtained from the qOCE probe during the indentation and analyzed for simultaneous quantification of reaction force (*F*) and depth resolved sample displacement (*d*(*z*)) that is considered as the material’s response to the excitation. Nevertheless, quantitative extraction of material properties remains challenging because the results of qOCE characterization (reaction force and displacement field established within the sample) not only depend on material properties, but also depend on the geometric boundary condition [15,16]. In this manuscript, we describe a method that achieves robust stiffness assessment using qOCE data (*F* and *d*(*z*)) and validate the method using experimental data. The capability to measure stiffness under different boundary conditions is crucial for intraoperative assessment of tumor margin in situ where the boundary condition is usually not known.

## 2. qOCE System

**Figure 1:**
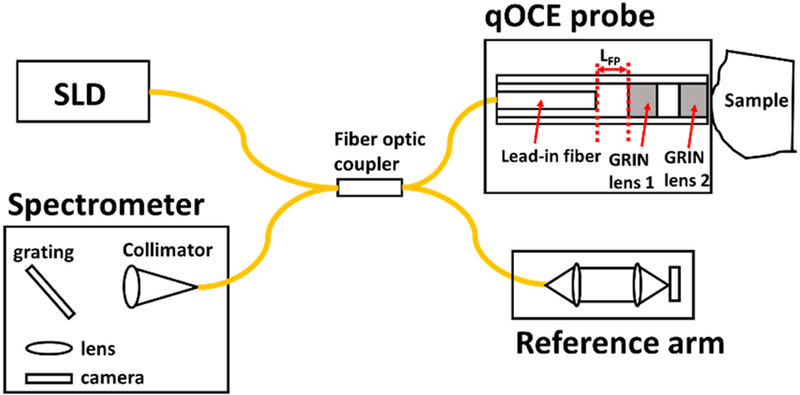
Configuration of the quantitative optical coherence elastography (qOCE) system.

Details about the qOCE technology have been described in our previous publications [13,14]. The qOCE system (Figure 1) operates on a spectral domain OCT (SD OCT) engine at 1310 nm based on a fiber-optic Michelson interferometer. The imaging system has a 2.5 mm imaging depth. The sample arm of the interferometer is interfaced with the novel qOCE probe that has a built-in Fabry–Perot (FP) force sensor and also acquires a signal from the sample underneath the probe. A common path OCT signal is generated due to the interference between optical fields reflected from two end facets of the FP cavity with a length of *L*_FP_. The signal peak (*I*_FP_) from the FP cavity is localized at *L*_FP_. With a force (*F*) excreted through the probe, the length of the FP cavity changes with the amount of ∆*L*_FP_ that is proportional to *F*: *F* = *k*∆*L*_FP_ where *k* is a parameter quantifying the stiffness of the probe shaft. We track the Doppler phase shift of $\left(\Delta \Phi_{\text{FP}}=\int_{t_{start}}^{t_{end}}a\tan[I_{FP}(t+\delta t)I_{FP}{}^{*}(t)]dt\right)$ where *t*_start_ and *t*_end_ indicate the starting and ending time of the indentation process and *δt* indicates the time interval between signals involved in Doppler analysis. With ∆Φ_FP_, we are able to quantify ∆*L*_FP_ (∆*L*_FP_ = (λ_0_∆Φ_FP_)/(4π) with λ_0_ indicating the central wavelength of the light source) and the force *F* (*F* = [*k*λ_0_/(4π)]∆Φ_FP_ = *α*∆Φ_FP_). Light exiting the probe also illuminates the sample. Light backscattered from the sample (***E***_s_) couplers back into the qOCE probe and interferes with reference light (***E***_r_) from the reference arm to generate a depth resolved OCT signal *I*_s_(*z*). Doppler analysis is applied to *I*_s_(*z*): ∆Φ_s_(*z*,*t*) = *a*tan[*I*_s_(*z*,*t* + *δt*)*I*_s_*(*z*,*t*)]. Depth resolved sample displacement is thus obtained: $d\left(z\right)=\frac{\lambda_{0}}{4\pi }\int_{t_{start}}^{t_{end}}\Delta \Phi \text{s}\left(z,t\right)dt$. By choosing the reference arm optical path length appropriately, the OCT signal for simultaneous probe deformation tracking (*I*_FP_) and tissue deformation tracking *I*_s_(*z*) can be multiplexed in the same Ascan without spatial overlap.

To generate qOCE data, the probe is translated to compress the sample and OCT signals are acquired during the compression process for the quantification of reaction force and the tracking of sample displacement. Notably, we performed M-mode scanning in this study. The probe acquires signals from the same spatial location over a period of time. Spatially resolved qOCE imaging is beyond the scope of this study. Imaging results can be found in our previous publications [12–14].

## 3. Sample Preparation

To validate our method for robust stiffness measurement, we fabricated polydimethylsiloxane (PDMS) phantoms [13]. These phantoms were made using a Sylgard 184 silicone elastomer base and Sylgard 184 silicone elastomer curing agent. Before curing, Titanium dioxide was added to provide light scattering. We adjusted the stiffness of the phantom by varying the base to curing agent ratio. The weight ratio between the elastomer base and curing agent was measured using a high precision balance (Sartorius practum 13–1 s). The mixture (PDMS base, curing agent and Titanium dioxide) was cured in a temperature controlled oven at 65 °C for 1 h. In this study, we fabricated phantoms with two different base-to-agent ratios (10:1 and 20:1), corresponding to stiffness of approximately 2.6 MPa and 1 MPa [17]. Phantoms with different thicknesses were also prepared.

## 4. Robust Stiffness Characterization Based on qOCE Measurement

The elastic modulus of a material (*E*) is defined as the ratio between the stress (*σ*) and the strain (*ε*): *E* = *σ*/*ε*. However, direct measurement of *E* is challenging because the stress and the strain are defined in the 3D space and have spatial variation. In a conventional indentation test for the measurement of stiffness, a material sample is compressed by an indenter. The reaction force (*F*) and indenter displacement (*h*) are measured. The elastic modulus is extracted using the *F*-*h* relationship based on simplified material models. With the assumption that an infinitely thick sample is compressed by a flat cylindrical indentor, the *F*-*h* relationship can be expressed as Equation (1) where *R* indicates the radius of the indentor and *ν* indicates the Poisson’s ratio [18]. However, the assumption of an infinitely thick sample is often not realistic. Therefore, the accuracy of stiffness assessment based on Equation (1) largely depends on the boundary conditions. In a modified model, a constant coefficient (*κ*) was introduced to take the indentor geometry (*R*) and sample thickness (*T*) into consideration (*κ* = *κ*(*R*/*T*)) (Equation (2)) [19]. However, the spatial variation of mechanical properties is often unknown during in situ tissue characterization:

(1) $$F=\frac{2RhE}{1-v^{2}}$$

(2) $$F=\kappa \left(\frac{R}{T}\right)\frac{2RhE}{1-v^{2}}$$

**Figure 2:**
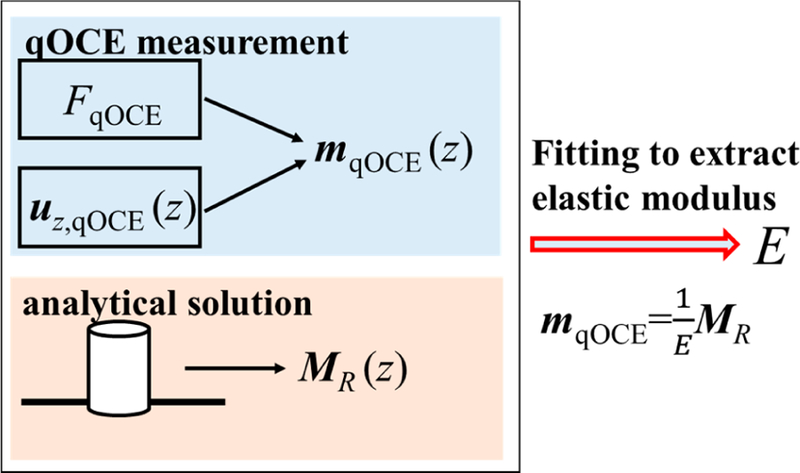
Extraction of elastic modulus using qOCE data.

To better utilize data obtained from qOCE measurement for robust stiffness assessment, we consider the analytical solution shown in Equation (3) for the axial displacement *d*(*z*) within an isotropic, linearly elastic sample indented by a flat cylindrical punch (qOCE probe) [20]. Here, *h* indicates the displacement of the indenter, *z* indicates the axial (depth) coordinate in the 3D space, Im indicates to take the imaginary part of a complex number and *i* is the imaginary unit. Assuming an incompressible sample (*ν* = 0.5), we take the ratio between *d*(*z*) (Equation (3)) and *F* (Equation (1)), and define the result as *m*_*qOCE*_(*z*) that is inversely proportional to the elastic modulus of the material (Equation (4)). Notably, *M*_*R*_ on the right-hand side of Equation (4) has an analytical expression shown in Equation (5). Therefore, the elastic modulus (*E*) can be extracted by fitting the linear model shown in Equation (6) where ***m***_*qOCE*_ is a vector obtained using qOCE data: ***m***_*qOCE*_ = *d*(*z*)/*F*_*qOCE*_, and vector ***M***_*R*_ = *M*_*R*_(*z*) derives from the above analytical solution (Equation (5)). The extraction of sample elastic modulus using qOCE data is illustrated in Figure 2:

(3) d(z)|x=0,y=0=hπ(1−v)Im[2(1−v)log(2z+2iR)−zz+iR],

(4) $$m_{qOCE}\left(z\right)=\frac{d\left(z\right)}{F}=\frac{1}{E}M_{R}\left(z\right),$$

(5) MR(z)=34πRIm[2(1−v)log(2z+2iR)−zz+iR],

(6) $$m_{qOCE}=\frac{1}{E}M_{R}$$

The above method (Figure 2) allows more robust assessment of sample stiffness. First, our method quantifies both geometric deformation and reaction force, which is essential for the measurement of elastic modulus (Equations (1) and (3)). In comparison, OCE that only measures sample deformation lacks the capability to quantify material properties. Secondly, a conventional indentation test measures the reaction force and the indenter displacement *h* that is significantly affected by the boundary condition of the measurement. Our method, on the other hand, uses local displacement (*d*(*z*)) of the sample rather than the global deformation. It is expected to result in improved robustness because *d*(*z*) in close proximity to the probe tip is largely determined by the probe geometry and the stress established at the contact plane. Other geometric factors, such as the sample thickness, play a much less significant role in determining the local displacement field immediately under the probe tip.

## 5. Results

### 5.1. Measurement Capabilities of qOCE

**Figure 3:**
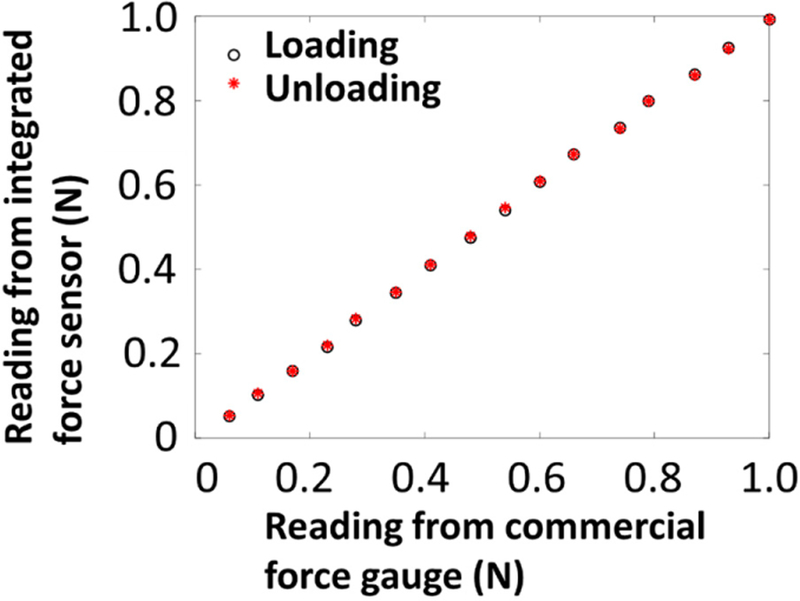
Force measurement of the qOCE instrument compared with the readings from a commercial force gauge.

We first validated the force sensing function of the qOCE system. We exerted force through the qOCE probe to the sensing tip of a commercial force gauge. Using Doppler phase shift (∆Φ_FP_) obtained from the OCT signal *I*_FP_ and the force reading from the force gauge, we extracted the coefficient *α* that converts a Doppler phase shift to a force value: F = *α*∆Φ_FP_. With the coefficient *α*, we exerted force through the qOCE probe in a loading (increasing force) and an unloading (decreasing force) process. The resultant force readings obtained from OCT data are shown in Figure 3, suggesting that the instrument allowed accurate force quantification in both the loading and unloading process.

**Figure 4:**
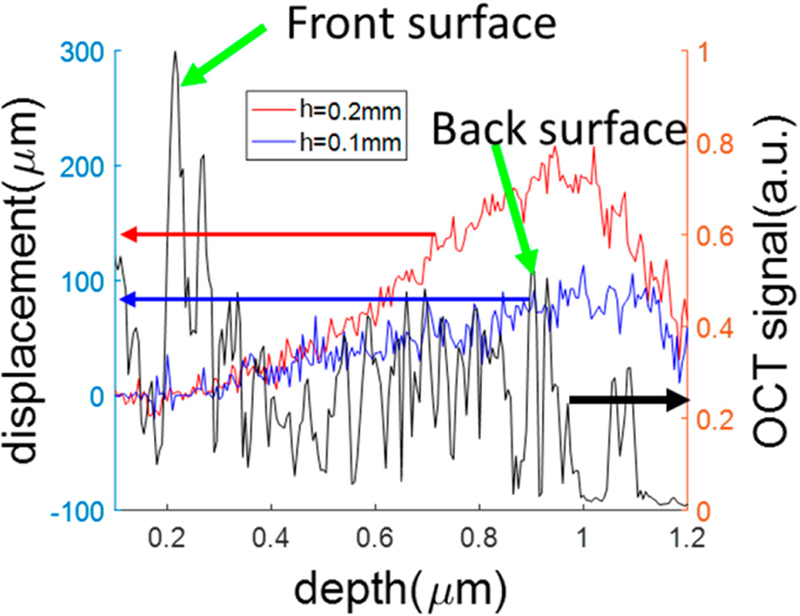
Magnitude optical coherence tomography (OCT) signal (black curve to the right axis), and depth resolved displacements extracted through Doppler analysis (red and blue curves) to the left axis.

To demonstrate the capability of our qOCE system in tracking depth resolved displacement (*d*(*z*)), we prepared a thin (*T* = 1 mm) elastic phantom. We placed the phantom on a flat, rigid surface, and compressed the phantom using the qOCE probe that was attached to a high precision linear motor to perform axial translation. The magnitude OCT signal obtained from the phantom is shown in Figure 4 (black curve with vertical axis on the right). The signal peak (green arrow) corresponding to the probe–sample interface can be easily identified. Through Doppler analysis, we also obtained depth resolved sample displacement (blue and red curves with vertical axis on the left). The blue curve was obtained when we translated the qOCE probe by 0.1 mm (*h* = 0.1 mm), and the red curve was obtained when we translated the qOCE probe by 0.2 mm (*h* = 0.2 mm). As shown in Figure 4, the displacement is approximately 0 at the probe–sample interface. Although the qOCE probe was translated axially by the linear motor, the probe–sample interface corresponded to a fixed optical path length. Therefore, the displacement extracted directly from the Doppler phase shift of the OCT signal started from 0 at the surface of the sample. Figure 4 also shows that the displacement (*d*(*z*)) increases gradually from 0 to *h* as *z* increases from 0 to *T*. It is worth mentioning that the magnitude of OCT signal and sample displacement were non-zero beyond the thickness of the sample (*T* = 1 mm) because photons experienced multiple scattering events. The ghost signal due to multiple scattering was not used in stiffness assessment. Although it is challenging to remove signals due to multiple scattering through digital filtering, multiple scattering artifacts do not compromise the signal quality in qOCE measurement when the thickness of the sample is sufficiently large.

### 5.2. Quantification of Elastic Modulus Using qOCE Data

**Figure 5:**
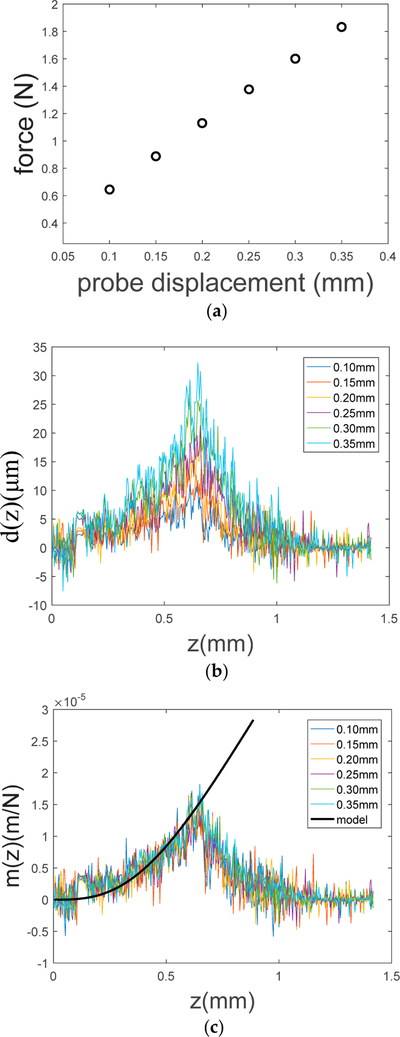
(**a**) reaction force corresponding to different probe displacement; (**b**) depth resolved sample displacement corresponding to different probe displacement; (**c**) *m*_*qOCE*_(*z*) obtained from experimental data and obtained analytically.

We performed qOCE measurement on a cylindrical PDMS phantom made with a 10:1 base-to-agent ratio (*E* = 2.6 MPa, 6 mm in thickness and 25 mm in diameter). We translated the probe at a speed of 0.1 mm/s and acquired an OCT signal at a 50 kHz Ascan rate. By translating the probe with different displacements, different reaction forces were obtained (Figure 5a) from the in-line fiber-optic force sensor. The displacement fields (*d*(*z*)) established within the sample were also acquired (Figure 5b). By normalizing *d*(*z*) with the corresponding reaction force *F*, *m*_*qOCE*_(*z*) was obtained for difference indenter displacements (Figure 5c). As indicated by Equations (4)–(6), *m*_*qOCE*_(*z*) remains the same for the same sample when the indenter is translated with different displacements. This can be observed in Figure 5c, where results obtained with different probe displacement are similar. Furthermore, with the expression of *M*_*R*_(*z*) shown in Equation (5) and the known elastic modulus of the material, we were able to synthesize a *m*_*qOCE*_(*z*) curve in Figure 5c (black, solid curve). It can be observed that the analytical solution (black curve) provides satisfactory approximation to the experimental data in a limited depth range (*z* < 0.6 mm). As the depth increases, *m*_*qOCE*_(*z*) extracted from qOCE data deviates from the analytical solution more significantly. This is because Doppler based displacement tracking becomes less accurate with signal attenuation as depth. Moreover, the actual displacement at a larger depth becomes more dependent on the measurement geometry. Therefore, the analytical solution (the black curve in Figure 5c) is valid within a limited depth range and it is crucial to choose an appropriate depth range of qOCE data for the assessment of sample stiffness.

**Figure 6:**
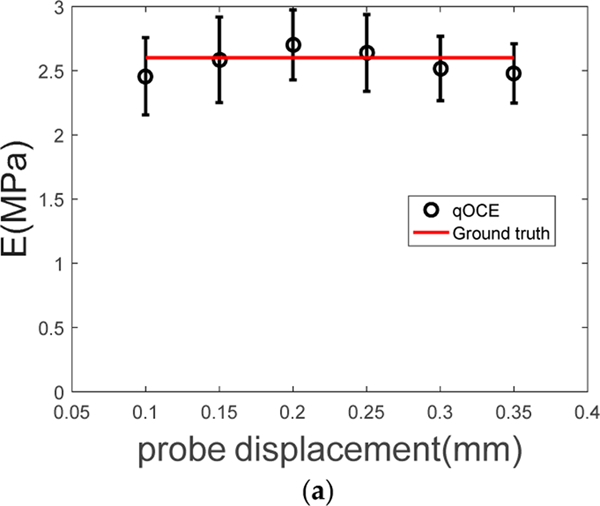

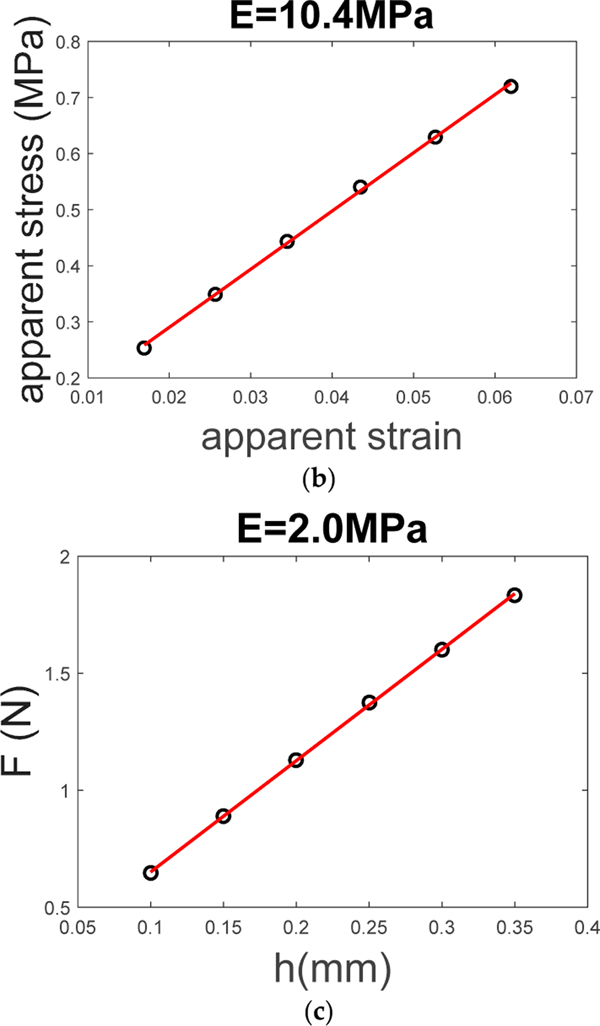
(**a**) elastic moduli obtained using *m*_*qOCE*_(*z*) in consistent with the known material stiffness; (b) the relationship between apparent stress and apparent strain results in an overestimation of stiffness; (c) the relationship between reaction force and indenter displacement results in an underestimation of stiffness.

Using experimentally acquired *m*_*qOCE*_(*z*) shown in Figure 5c, and *M*_*R*_(*z*) obtained analytically (Equation (5)), we performed linear fitting of Equation (6). The resultant elastic moduli are shown in Figure 6a where the error bars indicate 95% confidential interval of the fitting. The known stiffness of PDMS fabricated with a 10:1 base-to-agent ratio is also shown in Figure 6a as the red line, which is consistent with the results obtained by fitting qOCE data. For comparison, we also extracted the elastic modulus using the apparent stress (*σ*_a_ = *F*/*A* where *F* is the force reading from the in-line force sensor and *A* is the cross-sectional area of the qOCE probe) and the apparent strain (*ε*_a_ = *h*/*T* where *h* is the known probe displacement): *σ*_a_ = *Eε*_a_. By fitting this linear relationship using *σ*_a_ and *ε*_a_ at for different probe displacement (Figure 6b), we obtained an overestimated elastic modulus of 10.4 MPa. In addition, with the *F*-*h* data (Figure 6c), we also extracted the elastic modulus using Equation (1), which led to an underestimated elastic modulus of 2 MPa. The analysis based on apparent stress and apparent strain results in overestimation of stiffness because of the underlying assumption that the strain is uniformly distributed within the entire thickness of the sample. In fact, the deformation of the sample under compression limited to the volume in close proximity to the probe. The analysis based on *F*-*h* relationship underestimates the stiffness because the values of *F* and *h* are affected by the rigid surface at a finite depth on which the phantom is placed.

### 5.3. qOCE Assessment of Stiffness on PDMS Samples with Different Thicknesses

**Figure 7:**
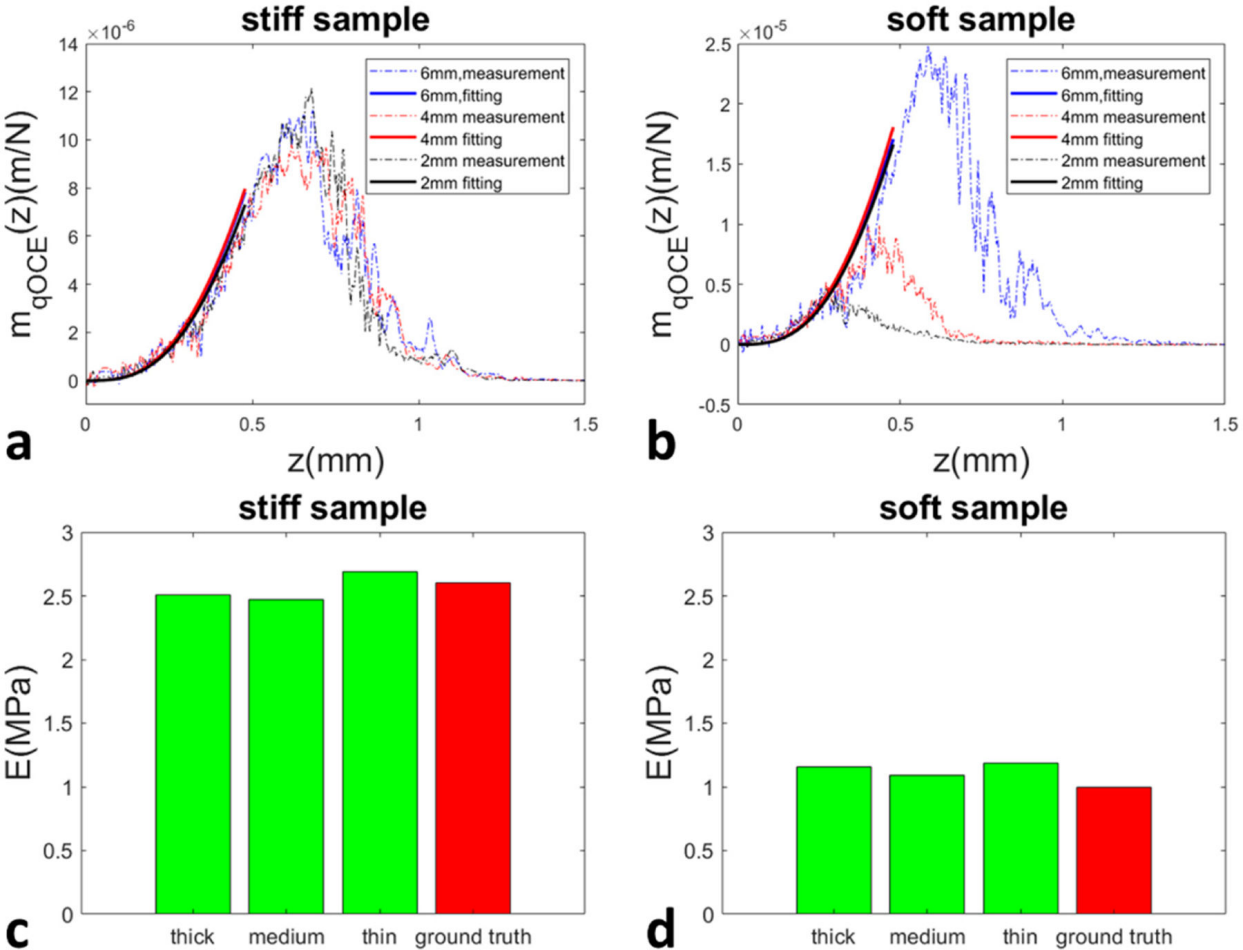
(**a**) experimental results (*m*_*qOCE*_(z)) from qOCE characterizations for stiff samples (*E* = 2.6 MPa) with different thicknesses, and curve fitting results; (**b**) experimental results (*m*_*qOCE*_(z)) from qOCE characterizations for soft samples (*E* = 1.0 MPa) with different thicknesses, and curve fitting results; (**c**) elastic moduli obtained from the stiff sample with large, medium and small thickness (green bars) in comparison with the value from literature (red bar); (**d**) elastic moduli obtained from the soft sample with large, medium and small thickness (green bars) in comparison with the value from literature (red bar).

We further demonstrated qOCE assessment of stiffness on stiff and soft PDMS samples that had different thicknesses. The stiff sample was made with a 10:1 base-to-agent ratio, corresponding to a stiffness of approximately 2.6 MPa. The soft sample was made with a 20:1 base-to-agent ratio, corresponding to a stiffness of approximately 1.0 MPa. The thicknesses of stiff and soft phantoms were 6 mm (thick), 4 mm (medium) and 2 mm (thin). To acquire qOCE data, we translated the probe axially to compress the sample. Reaction force and depth resolved sample displacement were obtained at the end of the compression process. We then normalized the displacement (*d*(*z*)) with the reaction force (*F*), as shown in Equation (4). The resultant *m*_*qOCE*_(*z*) curves for stiff and soft samples are shown in Figure 7a,b (dashed lines), respectively. Using the experimental data (*m*_*qOCE*_(*z*)) and the analytical *M*_*R*_(*z*) expressed in Equation (5), we were able to fit the linear model of Equation (6) to extract the elastic modulus. Notably, experimental data within a depth range of 0–330 µm was used in the fitting. With the elastic modulus (*E*) extracted, we show the fitting results (*M*_*R*_(*z*)/*E*) as solid lines in Figure 7a,b. The consistency between the experimental results and the fitted analytical function validates the effectiveness of the simplified material model for qOCE measurement within a limited depth range. The elastic moduli obtained from the stiff sample with large, medium and small thickness are shown in Figure 7c (green bars) in comparison with the value from literature (red bar). The elastic moduli obtained from the soft sample with large, medium and small thickness are shown in Figure 7d (green bars) in comparison with the value from literature (red bar). Figure 7c,d suggest that our method allows accurate stiffness assessment despite variations of sample thickness.

As shown in Figure 7a,b, the displacement field extracted from qOCE measurement does not have a linear dependency on depth. In other words, the strain field extracted is not spatially uniform. This is partially due to OCT signal attenuation with depth. As the depth increases, the OCT signal becomes overwhelmed by noise and the Doppler phase shift extracted from OCT signal cannot provide effective motion tracking. Regardless of OCT signal characteristics, a non-uniform strain field is established within the sample because the sample is compressed by an indenter with a finite dimension. The strain field is also affected by the geometry of the sample, which can be observed in Figure 7b. Moreover, the spatial variation of displacement/strain also depends on the mechanical properties (Figure 7a versus Figure 7b). Although the displacement field established within the deformed sample is complicated due to a wide range of factors, qOCE signal acquired from the volume immediately under the qOCE probe remains consistent and hence can be used to provide robust assessment of sample stiffness, suggesting that qOCE has the potential to perform robust in situ mechanical characterization where the boundary condition is not clearly known.

## 6. Conclusions and Discussion

In this manuscript, we describe a method that analyzes the data obtained from the qOCE system, in order to achieve robust stiffness assessment. We normalized the displacement field (*d*(*z*)) with the reaction force (*F*), and fit the result with an analytical model to extract the elastic modulus. Consistent stiffness was extracted from samples prepared in different thicknesses. With the improved robustness under different measurement geometry, we anticipate our method to enable the application of qOCE for margin assessment in breast cancer surgery.
